# Perceived Stress of Mothers, Harsh Discipline, and Early Childhood Mental Health: Insights from a Cross-Sectional Study in Marginalized Roma Communities

**DOI:** 10.3389/ijph.2024.1606721

**Published:** 2024-02-23

**Authors:** Daniela Filakovska Bobakova, Shoshana Chovan, Stanislava Van Laer

**Affiliations:** ^1^ Department of Health Psychology and Research Methodology, Faculty of Medicine, PJ Safarik University, Kosice, Slovakia; ^2^ Olomouc University Social Health Institute, Palacky University in Olomouc, Olomouc, Czechia; ^3^ Graduate School Kosice Institute for Society and Health, PJ Safarik University, Kosice, Slovakia

**Keywords:** mental health, perceived stress, early childhood, poverty, marginalized Roma communities, harsh discipline

## Abstract

**Objectives:** This study aims to compare the early childhood mental health of children from marginalized Roma communities (MRCs) in Slovakia with that of the majority and explore possible mediating pathways of mothers’ perceived stress and harsh discipline practices.

**Methods:** We used data from the first wave of the longitudinal RomaREACH study collected in 2021–2022. Two populations were included in the sample: 94 mother-child dyads from MRCs and 79 from the majority population (children aged 14–18 months). Data were analysed using linear regression, and mediation was tested using PROCESS Macro in SPSS.

**Results:** Belonging to MRCs vs. the majority, perceived stress of mothers and harsh discipline were found to be associated with early mental health problems in children. Perceived stress of mothers partially mediates the relationship between belonging to MRCs vs. majority and harsh discipline and harsh discipline partially mediates the relationship between perceived stress of mothers and the mental health of children.

**Conclusion:** Mothers from MRCs perceive more stress, which is associated with more frequent use of harsh discipline practices having a negative impact on the mental health of young children.

## Introduction

Anti-Roma racism and discrimination on interpersonal, as well as institutional and policy levels are perceived as root causes of health inequities in disadvantaged Roma population [1]. Although the Roma population in Slovakia is highly heterogeneous, approximately half of Roma live in more or less segregated localities referred to as marginalized Roma communities (MRCs) characterised by spatial and social distance from the majority population. People from MRCs often experience generational poverty, as well as limited access to education, employment, housing and various types of services, including healthcare [2], which causes material and financial hardship and leads to low quality of life [3, 4]. Centuries of oppression and continuous discriminatory practices make social mobility and benefiting from social capital for people living in generational poverty in MRCs hardly reachable [5]. The healthy development of children from MRCs is hindered by several interconnected factors acting at the individual, social and structural levels. Direct sources of early disadvantage can be found in the physical environment, the environment of relationships and nutrition [6]. Parental practices play a significant role in the mutual interactions of these factors and their impact on the healthy development of children.

The unfavourable socioeconomic situation of a family (regardless of ethnicity) can significantly influence parental practices through insufficient access to resources, as well as through the impact on the mental health of the parents. Families living in poverty face multiple forms of disadvantage and are disproportionately exposed to psychosocial stressors [7]. Chronic stress significantly contributes to psychological vulnerability [8] and affects social-emotional processes and cognitive control [9]. Repetti et al. (2002) emphasise the connection of low socioeconomic status (SES) with risky family characteristics, such as frequent family conflicts, a low level of support in relationships or the presence of violence in the family [10]. Other studies indicate that lower SES family environments are harsher and more repressive [11], with greater instability in daily family routines [12] and higher levels of chaos compared to higher SES environments [8]. Harsh discipline practices, such as physical punishment, yelling, criticism and controlling children’s behaviour, dilute children’s development and regulatory capacities [13].

Scarce research in the area of mental health of people living in MRCs suggests disparities in mental health between people living in MRCs and people from the majority population [14–16]. Olah, Biro and Kosa pointed out that the impact on mental health is strongly influenced by the living environment and socio-economic conditions in MRCs and not solely determined by ethnicity and emphasize the need to collect data on these aspects to avoid relying on self-reported Roma identity as an indicator of socioeconomic deprivation [14]. Research focusing on mental health in Roma children is rare and covers older age groups. Nevertheless, primary school Roma children were shown to have worse mental health outcomes compared to their non-Roma counterparts [17].

Thus, the study aims to compare the early childhood mental health of children from marginalized Roma communities in Slovakia with that of children from the majority and explore possible serial mediating roles of mothers’ perceived stress and harsh discipline practices.

## Methods

### Sample and Procedure

We used data from the first wave of the longitudinal RomaREACH (Research on Early Childhood in marginalized Roma communities) study. The data were collected in 2021–2022 in the Prešov and the Košice regions, which are regions with the highest share of marginalized Roma population in Slovakia. We included two populations in the sample: mother-child dyads from marginalized Roma communities and the Slovak majority population with children aged 14–18 months. Mothers of children were recruited during the child’s mandatory preventative paediatric visits, at the community centres and by spreading an invitation online via parenting groups on social media. Roma communities were selected from the Atlas of the Roma communities [4]. Mothers with low understanding and cognitive deficits, as well as mothers of prematurely born children were excluded from the sample. The final sample for the selected analyses consisted of 173 mother-child dyads with complete data on the domains of interest. Participants were recruited via paediatricians during regular preventive check-ups, via Roma health mediators and social workers directly in the communities and parental groups on social media. Data collection took place either in the cooperating outpatient departments, at community centres or in the households of respondents. Mothers filled out paper self-report questionnaires independently or with the assistance of the researchers and translation to Romani language by Roma health mediators if needed. Assisted self-administered interview adapted from other methods of collecting survey data, which seems to have the smallest impact on data reliability [18] was used to cope with low literacy of mothers from MRCs.

### Measures

Belonging to a **marginalized Roma community** was assessed based on the question “Are your closest neighbours mostly Roma?,” as well as by the administrators’ assessment of the respondent’s residence. The residence was assessed based on the Atlas of Roma communities—the sociographic mapping conducted by the Office of the Government Plenipotentiary for the Roma Communities in cooperation with the Institute for Work and Family Research undertaken in 2019 [4].

**The degree of poverty** was expressed with three different measures: mothers’ education (elementary/secondary/university), presence of running water in the household (yes/no) and billing problems [19]. Billing problems were assessed based on five items measuring weather the household has encountered problems paying for some of the expenses: 1. Rent; 2. Water, electricity, gas; 3. Food, clothing; 4. Loan repayments; 5. Healthcare costs in the past year. Participants could answer either *yes* or *no*. Sum-score ranged from 0 to 5 (Cronbach’s *α* = 0.74).

**Perceived stress** was assessed using the Shortened Perceived Stress Scale (PSS-4) [20] based on four items, including questions on how unpredictable, uncontrollable and overloaded respondents found their lives in the past month. The answers range from *never* = 1 to *very often* = 5. Sum-score ranged from 4 to 20 (Cronbach’s *α* = 0.55). A higher score indicates higher levels of perceived stress.

**Harsh discipline** was assessed using the harsh discipline domain of the comprehensive early childhood parenting questionnaire (CECPAQ), which includes data on verbal, physical and psychological control [21]. The response categories indicated how often parents showed the described behaviour and ranged from *does not concern me* = 0 to *always* = 5. Sum-score ranged from 0 to 60 (Cronbach’s *α* = 0.91). A higher score indicates more frequent use of harsh discipline practices.

Early childhood **Mental health** was measured using the Mental Health subscale of the Caregiver Reported Early Development Instrument (CREDI), which was designed to measure development in children under 3 years of age [22] and to be used in low resource settings [23]. The mental health subscale captures early symptoms of children’s mental health, including behaviours related to aggression, anxiety and distress [24]. Sum-score ranged from 0 to 9 (Cronbach’s *α* = 0.64). A higher score indicates more mental health problems.

**Background variables** included the child’s sex and maternal age.

### Statistical Analyses

First, we described the sample using descriptive statistics. Differences between the two groups (mothers from MRCs vs. mothers from the majority population) were assessed using the Mann–Whitney *U*-test and Chi-squared test. Mann–Whitney *U*-test was used as the distribution was not normal according to Kolmogorov-Smirnov and Shapiro-Wilk tests. Next, we explored the association of belonging to MRCs vs. the majority, perceived stress of mothers and harsh discipline with the early mental health of children, adjusted for the child’s sex and maternal age using linear regression on 1,000 bootstrapped samples, since the residuals of the models were not normally distributed. We assessed the mediating role of harsh discipline on the relationship between belonging to MRCs vs. majority and the mental health of children and between perceived stress of mothers and the mental health of children. We also assessed the mediating role of the perceived stress of mothers on the relationship between belonging to MRCs vs. majority and harsh discipline. Finally, we conducted serial mediation analyses to assess whether the perceived stress of mothers and harsh discipline serially mediate the differences in the early mental health of children between the two groups. *p*-value lower than 0.05 was considered significant. Statistical analyses were performed using IBM SPSS 23 for Windows. Simple and serial mediations were tested using PROCESS Macro in SPSS Model 6 on 5,000 bootstrap samples.

## Results

### Description of the Sample

Table 1 shows the descriptive statistics for mothers from MRCs and mothers from the majority population. Significant differences between the groups were found in all the explored measures of interest (perceived stress of mothers, harsh discipline and early mental health of children).

**TABLE 1:** Description of the sample [Research on Early Childhood in marginalized Roma communities (RomaREACH) study, Slovakia, 2021–2022].

	Majority (*n* = 79)		MRCs (*n* = 94)		Total (*n* = 173)		*p*-value
	*N*	(%)	*N*	(%)	*N*	(%)	
Child’s sex							ns
Boy	33	41.8	45	47.9	78	45.1	
Girl	46	58.2	49	52.1	95	54.9	
Mothers’ education							***
Elementary	1	1.3	73	77.7	74	42.8	
Secondary	18	22.8	21	22.3	39	22.5	
University	60	75,9	0	0	60	34.7	
Running water	78	98.7	48	51.1	126	72.8	***
	Mean (SD)		Mean (SD)		Mean (SD)		
	Median (IQR)		Median (IQR)		Median (IQR)		
Age of the mothers (in years)	32.18 (3.90)		24.93 (5.87)		28.24 (6.22)		***
	32.00 (30.00–35.00)		24.00 (20.00–29.00)		29.00 (23.00–33.00)		
Billing problems	0.14 (0.64)		1.03 (1.30)		0.62 (1.14)		***
	0.00 (0.00–0.00)		1.00 (0.00–2.00)		0.00 (0.00–1.00)		
Perceived stress of mothers	7.85 (2.85)		9.54 (3.14)		8.77 (3.12)		***
	7.00 (6.00–9.00)		10.00 (7.00–11.00)		9.00 (6.00–11.00)		
Harsh discipline	17.47 (5.30)		22.22 (10.93)		20.05 (9.11)		*
	16.00 (14.00–19.00)		19.50 (13.00–28.25)		18.00 (14.00–23.00)		
Mental health of children	2.24 (1.58)		4.43 (1.87)		3.43 (2.05)		***
	2.00 (1.00–3.00)		4.00 (3.00–5.00)		3.00 (2.00–5.00)		

*p < 0.05, **p < 0.01, ***p < 0.001.

### Predictors of Poor Mental Health

Table 2 shows the results of the linear regression analyses. Belonging to MRCs vs. the majority, perceived stress of mothers and harsh discipline were found to be associated with the early mental health of children.

**TABLE 2:** Predictors of poor mental health [Research on Early Childhood in marginalized Roma communities (RomaREACH) study, Slovakia, 2021–2022].

	Mental health B (CI)
MRCs	2.19 (1.69–2.72)***
Perceived stress of mothers	0.21 (0.12–0.30)***
Harsh discipline	0.08 (0.05–0.12)***

*p < 0.05, **p < 0.01, ***p < 0.001.

### Mediation Analysis

**FIGURE 1:**
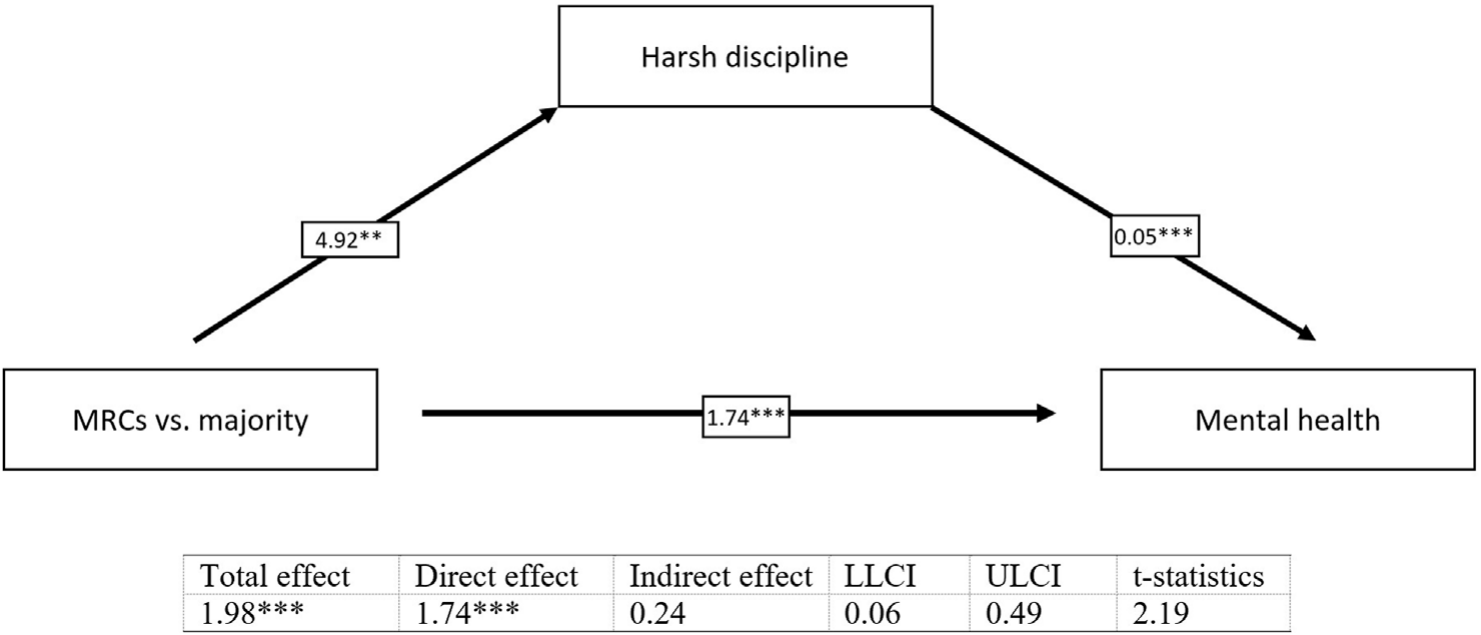
Mediation of harsh discipline on the relationship between belonging to MRCs vs. majority and mental health of children, adjusted for child’s sex and maternal age [Research on Early Childhood in marginalized Roma communities (RomaREACH) study, Slovakia, 2021–2022, *n* = 173].

**FIGURE 2:**
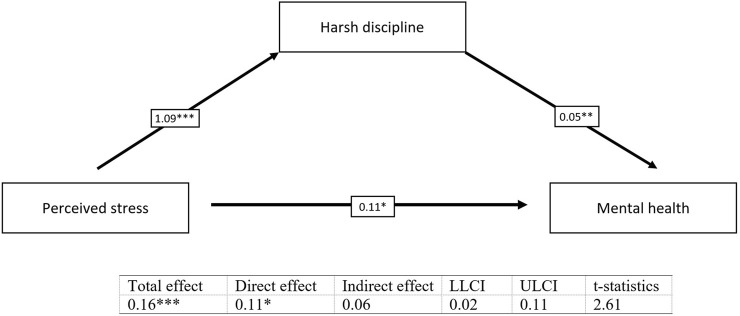
Mediation of harsh discipline on the relationship between perceived stress of mothers and mental health of children, adjusted for child’s sex and maternal age [Research on Early Childhood in marginalized Roma communities (RomaREACH) study, Slovakia, 2021–2022, *n* = 173].

**FIGURE 3:**
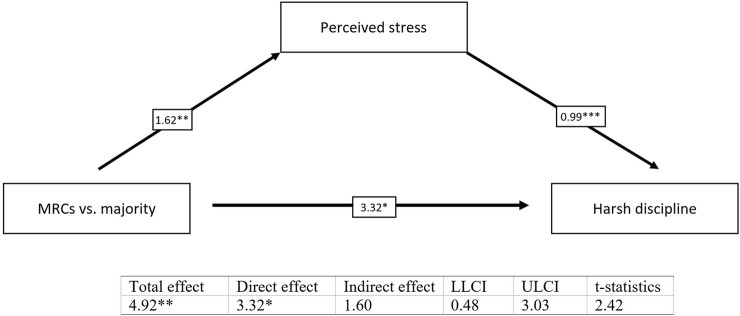
Mediation of perceived stress of mothers on the relationship between belonging to MRCs vs. majority and harsh discipline, adjusted for child’s sex and maternal age [Research on Early Childhood in marginalized Roma communities (RomaREACH) study, Slovakia, 2021–2022, *n* = 173].

**FIGURE 4:**
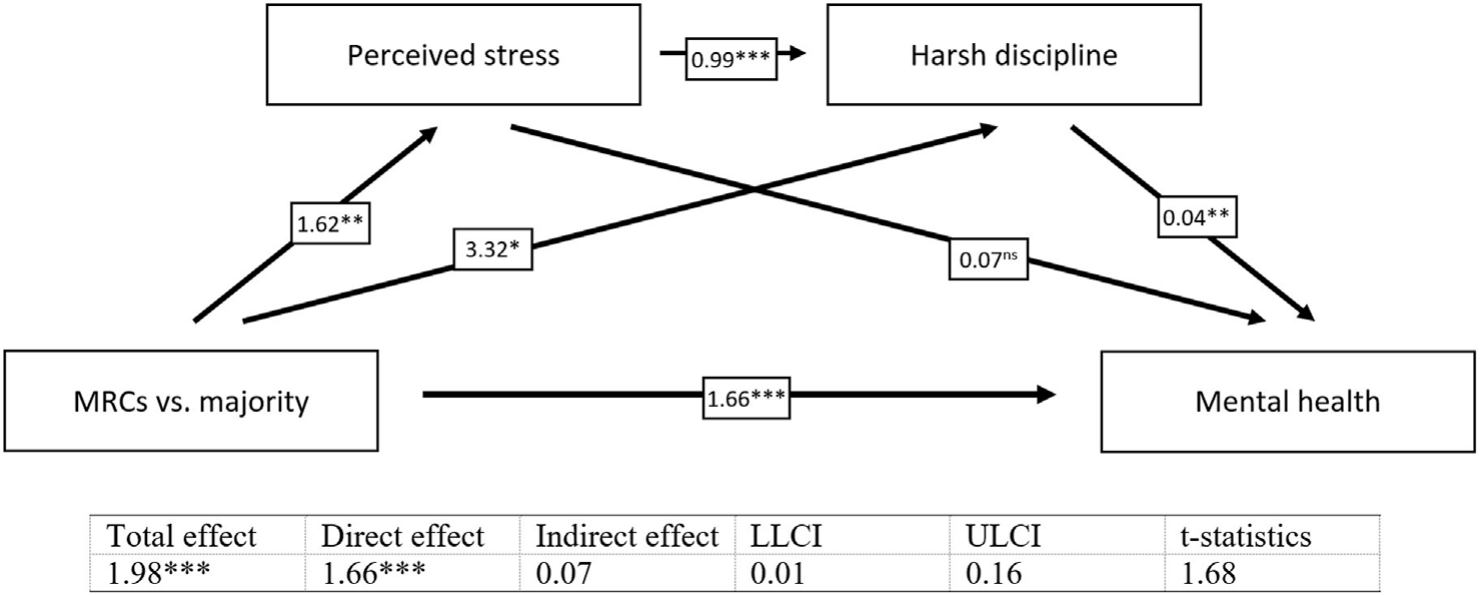
Serial mediation of perceived stress of mothers and harsh discipline on the relationship between belonging to MRCs vs. majority and the early mental health of children, adjusted for child’s sex and maternal age [Research on Early Childhood in marginalized Roma communities (RomaREACH) study, Slovakia, 2021–2022, *n* = 173].

The results revealed significant indirect effects in all tested simple mediation models. Harsh discipline partially mediates the relationship between belonging to MRCs vs. the majority and the mental health of children (*b* = 0.24, *t* = 2.19) (Figure 1) and the relationship between perceived stress of mothers and the mental health of children (*b* = 0.06, *t* = 2.61) (Figure 2). We found also a significant indirect effect of belonging to MRCs vs. the majority on the harsh discipline through perceived stress of mothers (*b* = 1.60, *t* = 2.42) (Figure 3). The indirect effect of belonging to MRCs vs. the majority on the early mental health of children through perceived stress of mothers and harsh discipline was not confirmed due to low t-statistics (*b* = 0.07, *t* = 1.68) although the confidence interval of indirect effect is not passing through the zero (CI = 0.01; 0.16) and coefficients of direct and indirect effects indicate complimentary serial mediation. The direct effect of belonging to MRCs vs. the majority on the early mental health of children in the presence of mediators was found to be significant (*b* = 1.66, *p* < 0.001). The mediation summary is presented in Figure 4.

## Discussion

We found that the mental health of children from MRCs is significantly worse compared to their better-off peers from the majority population. Mothers of children from MRCs perceive higher levels of stress and use harsh discipline practices more often than mothers from the majority. Both, the effect of belonging to MRCs vs. the majority and the effect of the perceived stress of mothers on the early mental health of children are mediated via harsh discipline. Moreover, the perceived stress of mothers mediates the differences between mothers from MRCs and the majority in harsh discipline. However, serial mediation of perceived stress of mothers and harsh discipline on the relationship between belonging to MRCs vs. majority and the early mental health of children was not confirmed.

Although the preferred style of parenting is responsive parenting, in the context of the psychosocial disadvantage of MRCs it might be hard to follow one’s own ideals about this [25–27]. Moreover, mothers from MRCs may lack social and cultural capital as well as competencies; therefore, they might not be aware of specific parental strategies to fulfil the aforementioned parenting style. This might result from the social distance from the majority population caused by marginalisation, leading to low education and awareness of mothers from MRCs. Anti-Roma racism recognized as a root cause of inequities [1] and unfavourable circumstances accumulated in MRCs such as low educational levels, high unemployment rates, poverty and poor living conditions [19] that have negative effects on each aspect of the everyday lives of their residents, including psychological vulnerability, chronic stress and the mental health of parents [7, 8]. Public discourse attributing parental challenges in MRCs solely to individual shortcomings overlooks the structural roots and the intricate challenges arising from generations of poverty, highlighting the significance of considering social determinants of health [28] in addressing these issues. Persistent anti-Roma racism in Europe, perpetuated through stereotypical, racist, and discriminatory discourse, hinders the success of policies targeting MRCs [29]. It is, therefore, necessary to view our findings through this lens.

Our findings suggest that children from MRCs have worse mental health compared to children from the majority. This is in line with previous studies conducted in disadvantaged settings with children living in poverty, which showed similar differences compared to better-off children [17, 30]. The evidence is even more robust in longitudinal studies demonstrating the detrimental effect of childhood poverty on mental health in adolescence and adulthood [31–33]. Our results indicate that living in MRCs characterised by poverty affects the mental health of children as early as in the first years of life.

Harsh discipline, which is more prevalent in MRCs, plays an important role in early childhood mental health outcomes. More frequent use of harsh discipline practices is associated with worse mental health outcomes in children and mediates the relationship between belonging to MRCs vs. the majority and the early mental health of children. A possible interpretation is that the higher prevalence of the use of harsh discipline practices in MRCs can be explained by the accumulation of disadvantage and poverty. This is in line with previous studies showing that a lack of resources is associated with harsh parenting practices [34–37] and that harsh parenting mediates the relationship between poverty and mental health outcomes in children [10, 30, 38].

Higher levels of perceived stress are more prevalent among mothers from MRCs and are related to more frequent use of harsh discipline practices, which in turn influence the mental health of their young children. An explored mediating pathway suggests that living in poverty leads to higher levels of stress in caregivers, which is associated with disrupted parenting practices [39, 40] that affect the mental health of young children [30]. Nevertheless, since all tested mediations revealed only partial mediation effects and the serial mediation was not fully confirmed other factors likely play a part in the relationship belonging to MRCs vs. majority and early mental health of children. Moreover, mothering, although a deeply individual experience, is profoundly influenced by structural forces such as the welfare state, exclusion, marginalization, and discrimination [41]. Women from ethnic minorities experiencing financial hardship construct their motherhood in response to the chronic stress of poverty, discrimination, and long-term structural processes that disadvantage them [42–45].

### Strengths and Limitations

Our study is the first to focus on mental health in such young disadvantaged Roma children (14–18 months old) and demonstrates possible pathways through which living in poverty of MRCs may influence perceived stress in mothers and the use of harsh discipline practices that have a negative impact of early mental health of their children. Some limitations need to be mentioned. The size of the sample and a cross-sectional design lowers the power of the study to detect all effects and differences and does not allow conclusions about causality. As we did not include either Roma mothers with higher socioeconomic status outside of MRCs or mothers from a majority living at a disadvantage, the two groups compared are very distinct and represent opposite sides of the socioeconomic spectrum. Furthermore, self-reported data are prone to social desirability, which may cause an underestimation of the use of harsh discipline practices in mothers and the mental health problems of children. Data from the mothers living in MRCs were mostly collected via assisted self-administered interview in Slovak language and data from mothers from majority via self-reported questionnaires. The reason for this was to cope with illiteracy, which we considered to be a more serious source of non-response and bias than using two different types of administration which has shown good reliability of data [18].

### Implications for Practice, Policy and Future Research

An appropriate parental approach is vital, especially in the first years after the birth of the child, which are characterised by a greater degree of physical and psychological dependence of young children on their caregivers [46]. It is also particularly important for children living in poverty, who are exposed to an increased risk of health problems and delays in psychomotor development [47]. Investments in early childhood development have the greatest benefit for the most disadvantaged children and a long-term equalising effect. However, interventions must not only address the survival and physical health of children at an early age but also their healthy psychosocial development [28]. Poverty and social exclusion are likely contributing significantly to experiencing stress in mothers and having adverse effects on parenting practices [39, 40]; thus, supporting families in providing a nurturing environment for their children and providing opportunities to strengthen parenting skills might help to better the mental health of young children and influence their health outcomes later in life. Previous research has shown that addressing poverty in MRCs to support early childhood development is necessary but not easily implemented due to unfavourable public discourse and lack of political will [2]. These arguments can also be used in advocating for enhancement of living conditions in MRCs, focusing on sources of stress that stem from poverty. Moreover, better access to early childhood education and care services and implementation of interventions focusing on the transfer of cultural capital via educational community programmes working with mothers from MRCs fostering parenting skills and childcare might mitigate the effect of poverty on healthy early childhood development, including mental health [48]. Programmes supporting parents in their care of children are considered a preventive strategy with the potential to mitigate the long-term negative impact of poverty on child health and development [49].

On the policy level, continuous efforts are needed to tackle discrimination and advocate for equity of the Roma minority in the field of access to housing, education and the labour market. Implementation of the necessary measures to tackle poverty in MRCs will require a change in social and political discourse and the courage of politicians and institutions to introduce measures that require larger investments and effective use of European funds, despite such measures probably not being popular in the eyes of a portion of the public and will not lead to political points being won.

Further research on a larger scale should confirm and further explore the factors and pathways of worse mental health in children from MRCs. A longitudinal study could establish the causal pathways between the predictors and parenting practices and developmental outcomes. Interventions supporting parenting skills, promoting parental mental health and focusing on early childhood development that are already in place should be evaluated, improved and upscaled.

### Conclusion

The poor quality of interactions between children and mothers living in poverty of MRCs are the consequences of a disorganised and stressful psychosocial environment and are associated with poor mental health of young children. Mothers from MRCs perceive less control over their lives and a lower ability to cope with everyday problems compared to mothers from the majority, and this stress is associated with frequent use of harsh discipline practices having a negative impact on the mental health of young children. Besides the necessity to tackle poverty in MRCs, programmes supporting parents and focusing on early childhood development have the potential to mitigate the long-term negative impact of poverty on child health and development.
